# Momentary Mediational Associations Among Affect, Emotion Dysregulation, and Different Types of Loss of Control Eating Among Adults With Binge Eating Disorder

**DOI:** 10.1002/eat.24415

**Published:** 2025-03-15

**Authors:** Kelly A. Romano, Carol B. Peterson, Glen Forester, Joseph A. Wonderlich, Stephen A. Wonderlich, Scott E. Engel, Ross D. Crosby

**Affiliations:** ^1^ Department of Psychiatry and Behavioral Sciences University of Minnesota Medical School Minneapolis Minnesota USA; ^2^ Center for Biobehavioral Research Sanford Research Fargo North Dakota USA; ^3^ Department of Psychiatry and Behavioral Science University of North Dakota School of Medicine and Health Sciences Fargo North Dakota USA

**Keywords:** binge eating disorder, disordered eating behaviors, eating disorders, ecological momentary assessment, emotion dysregulation, loss of control eating

## Abstract

**Objective:**

Few studies have directly assessed the mechanistic role of transdiagnostic self‐regulatory factors that are theorized to promote core disinhibited disordered eating behaviors that characterize binge eating disorder (BED) in the natural environment, such as emotion dysregulation. The present study used ecological momentary assessment (EMA) to address this research gap by examining whether: (1) emotion dysregulation mediated associations between negative and positive affect and loss of control (LOC) eating at the within‐person level; (2) these associations varied across distinct LOC eating dimensions.

**Method:**

Adults with BED (*N* = 107; *M*_age_ = 39.87, SD = 13.35) responded to six surveys per day for a 7‐day EMA period. Multilevel structural equation models examined whether momentary emotion dysregulation mediated momentary associations between negative and positive affect, and different LOC eating outcomes (“general” [subjective experience of] LOC while eating; difficulties resisting eating; difficulties stopping eating after starting; feeling driven/compelled to eat; not paying attention to one's eating; feeling disconnected while eating [e.g., numb, zoned out]).

**Results:**

Experiencing a sequential worsening of negative affect and, in turn, emotion dysregulation over a day mapped onto higher levels of certain LOC eating outcomes (“general” LOC eating, difficulties resisting eating, driven/compelled to eat, disconnected while eating) but not others (difficulties stopping eating, not paying attention to one's eating). All momentary mediational pathways involving positive affect as a predictor were not significant.

**Discussion:**

These findings support emotion dysregulation as a mechanistic process that can precipitate certain types of LOC eating in daily life and may be leveraged to improve BED theory, research, and real‐time interventions.

**Summary**

- The present findings suggest that when adults with binge eating disorder experience a sequential worsening of their negative (but not positive) affect and, in turn, emotion dysregulation over the course of the day, they engage in more severe levels of distinct types of loss of control eating later that day.
- These results also provide insight that may help strengthen eating disorder theories, research, and inform future eating disorder interventions.

## 1 Introduction

Binge eating disorder (BED) is a debilitating mental health condition that is associated with elevated mortality risk and morbidity (Hambleton et al. 2022; Santomauro et al. 2021; van Hoeken and Hoek 2020). Further, over half of individuals with BED relapse or remain symptomatic following empirically supported BED treatments (Linardon 2018; Sala et al. 2023). Despite these adverse health impacts and suboptimal treatment outcomes, research on mechanisms that are theorized to contribute to BED maintenance and underlie individuals' engagement in disordered eating behaviors (DEBs) in their natural environments (e.g., at home, work/school) remains poorly understood. The limited research in this area serves as an important missed opportunity that, if addressed, may provide insight into mechanistic processes that may be leveraged for testing as treatment targets in novel real‐time interventions and potentially improve BED outcomes.

### 1.1 Transdiagnostic Self‐Regulatory Processes

Several BED theories posit that transdiagnostic self‐regulatory processes—such as affect and emotion dysregulation—promote recurrent engagement in core disinhibited DEBs that characterize BED (e.g., loss of control [LOC] eating, binge eating) and contribute to BED maintenance (Hawkins and Clement 1984; Heatherton and Baumeister 1991; Mason et al. 2021). Specifically, these theories posit that individuals attempt to distance themselves or “escape,” from adverse affective states that emerge on a moment‐to‐moment basis (e.g., high momentary negative affect, low momentary positive affect) by engaging in DEBs that can promote temporary affective improvements. In turn, these post‐DEBs affective improvements can promote recurring DEBs and, over time, a negatively reinforced cycle that maintains BED (Hawkins and Clement 1984; Heatherton and Baumeister 1991; Mason et al. 2021). Prior ecological momentary assessment (EMA) research has generally supported these hypotheses by demonstrating that individuals' negative affect levels progressively increase and positive affect levels decrease in the hours preceding LOC eating, overeating, and binge eating, and subsequently improve following the occurrence of these DEBs (Berg et al. 2017; Schaefer et al. 2020; Wonderlich et al. 2022). Further, preliminary EMA research has also shown that greater momentary positive emotional eating (i.e., overeating in response to positive affect) significantly predicted greater momentary overeating among community‐based and college women (Sultson et al. 2017).

Complementing this EMA research supporting these within‐person (momentary, daily) processes, a large primarily between‐person (cross‐sectional) evidence‐base that attests to trait‐level processes indicates that it is not only dynamic moment‐to‐moment changes in the intensity or experience of affect (i.e., negatively and positively valenced emotional experiences) that promotes recurrent LOC eating and related disinhibited DEBs but emotion dysregulation (i.e., varied difficulties modulating emotions and behaviors) also upholds a vital role in this process (Lavender et al. 2015, 2014; Prefit et al. 2019). Although this research supports emotion dysregulation as a robust correlate of and mechanism that promotes BED pathology at the between‐person level (Lavender et al. 2015, 2014; Prefit et al. 2019), little is known about whether these associations manifest at the within‐person level based on research using ambulatory assessment (e.g., EMA, daily diaries). This research gap is important, as emotion dysregulation is a fluctuating process that changes on a moment‐to‐moment basis due to intrapersonal (e.g., affect intensity, negative urgency), interpersonal (e.g., social support availability), and environmental (e.g., context‐specific social norms) factors (Gratz and Roemer 2004; Gross 1998a; Gross 1998b; Lavender et al. 2015, 2014) that cannot be fully captured by between‐person methods. Instead, ambulatory methods are designed to assess dynamic processes in real‐time and can answer novel research questions, such as “*at times when* individuals experience worse affect and emotion dysregulation than usual—regardless of their trait‐levels—do they use DEBs *at those times* and/or *later that day*?” (Shiffman et al. 2008). Consequently, research using ambulatory methods can determine how dynamic changes in emotion dysregulation that emerge in the natural environment over a day promote individuals' DEBs and explain affect‐DEBs associations.

### 1.2 Within‐Person Research

To our knowledge, only three prior studies have used ambulatory methods to examine within‐person associations among affect, emotion dysregulation, and DEBs. Two of these studies showed that within‐person emotion dysregulation did not moderate momentary or daily associations between negative affect and either binge eating or emotional eating in one EMA study with adults with BED (Svaldi et al. 2019) or in one daily diary study of community‐based twin women (Mikhail et al. 2022). However, the daily diary study showed that greater emotion dysregulation (operationalized as maladaptive strategy use) on a given day was associated with greater binge eating and purging the subsequent day via greater next‐day increases in negative—but not positive—affect (Mikhail et al. 2024). Notably, this study did not examine whether daily emotion dysregulation mediated any daily affect‐DEBs associations, despite evidence that emotion dysregulation upholds a central role in the experience, modulation, and expression of affect and, consequently, is theorized to serve as a core mechanism underlying associations between affect and BED pathology (Gratz and Roemer 2004; Lavender et al. 2015). Given the mixed results from this very small literature, additional research is needed to improve the understanding of how emotion dysregulation may promote DEBs on a day‐to‐day basis.

Two research gaps warrant particular attention. First, LOC eating (i.e., the subjective experience of losing control while eating) is a clinically, theoretically, and diagnostically important DEB that is a core component of objective binge eating behavior; that is, overeating episodes (i.e., consuming an objectively large amount of food, given the circumstances) with co‐occurring LOC (American Psychiatric Association [APA] 2022; World Health Organization [WHO] 2019). It is well established that greater LOC eating severity—independent of the amount of food one consumes—is robustly and uniquely associated with greater eating pathology, clinical impairment, and adverse psychophysiological health outcomes (Goldschmidt 2017; Goldschmidt et al. 2012; Mond et al. 2010). Consequently, LOC eating is commonly conceptualized as a marker of greater BED severity and poorer health (Bottera and De Young 2024; Goldschmidt 2017; Latner et al. 2014). Notably, LOC eating is a heterogeneous construct with different behavioral (e.g., losing control while eating, difficulties resisting eating) and cognitive (e.g., feeling dissociated/disconnected while eating) dimensions that have exhibited unique associations with eating disorder and general psychopathology in between‐person research (e.g., Latner et al. 2014; Stefano et al. 2016). For example, in two between‐person (cross‐sectional) studies with samples of college students with elevated ED risk, positive associations between behavioral dimensions of LOC eating and global ED pathology, DEBs (e.g., binge eating frequency), ED‐related psychosocial impairment, and feelings of distress were generally stronger than those identified for cognitive/dissociative dimensions, which in turn were stronger than the respective positive associations that were identified for positive/euphoric LOC eating dimensions (e.g., feeling a sense of relief or a euphoric “rush” while engaging in LOC eating; Latner et al. 2014; Stefano et al. 2016). The variations that were identified in the strength of these associations as a function of the LOC eating dimensions under consideration may stem from the notion that behavioral aspects of LOC eating may be easier to notice by individuals, and therefore easier to report, than more intrapersonal cognitive or euphoric components that may operate at a more covert level and can be more difficult for individuals to recognize (Latner et al. 2014; Stefano et al. 2016). Although these data provide important insight into how these processes emerge at the between‐person level, as a notable research gap, no research has used EMA to examine how varied LOC eating dimensions may be differentially promoted in the natural environment by core transdiagnostic self‐regulatory factors, such as affect and emotion dysregulation.

Second, to our knowledge, no research has examined whether momentary emotion dysregulation mediates momentary associations between negative and positive affect relative to DEBs (including LOC eating) among individuals with BED. As a proxy, in one EMA study with young women (not adults with BED) emotion dysregulation dimensions were assessed as momentary mediators of momentary associations between negative and positive affect relative to LOC eating, overeating, and dietary restriction (Romano et al. 2024). Results showed that one facet of women's emotion dysregulation—momentary difficulties with emotional and behavioral modulation—mediated momentary associations between negative affect and LOC eating and overeating, but not dietary restriction (Romano et al. 2024). Notably, in this prior study, LOC eating was assessed using one general single‐item that captured whether participants “felt as though they could not stop eating after starting.” It remains unclear whether these results: (1) extend to adults with BED who experience disproportionate physiological and psychosocial health consequences from their eating disorder symptoms (Hambleton et al. 2022; Linardon 2018; Sala et al. 2023; Santomauro et al. 2021; van Hoeken and Hoek 2020), rather than young women only; (2) and, vary across different LOC eating dimensions that have been suggested to upload distinct roles in the onset and maintenance of BED pathology, based on existing theoretical and conceptual knowledge, clinical observations, and between‐person (e.g., cross‐sectional) research (see above; e.g., Latner et al. 2014; Stefano et al. 2016), rather than a general broad conceptualization of LOC eating only. Addressing these research gaps may identify variations in how these associations manifest in everyday life, help increase the precision of real‐time BED interventions, and enhance mechanistic BED research and theories.

### 1.3 Study Purpose

The present study aimed to address these research gaps by examining: (1) whether emotion dysregulation mediated associations between negative and positive affect relative to LOC eating at the momentary level; (2) variations in these associations across different LOC eating dimensions. Of note, recent research has shown that greater momentary negative (but not positive) affect served as a momentary mediator of an association between greater momentary difficulties with emotional and behavioral modulation and greater general LOC eating (with LOC eating operationalized as a general perception that participants felt that they could not stop eating after starting via a single survey item), but not overeating or dietary restriction, in an EMA study with young women (Romano et al. 2024). Also, daily negative (but not positive) affect served as a mediator of daily associations between emotion dysregulation strategy use (i.e., global maladaptive emotion regulation strategy use) relative to binge eating and purging among community‐based twin women in a daily diary study (Mikhail et al. 2024). However, neither prior study examined distinct LOC eating dimensions as outcomes. Consequently, the directionality of these within‐person mediational associations (affect → emotion dysregulation → LOC eating versus emotion dysregulation → affect → LOC eating) and variations that may emerge for different LOC eating types are unclear. Given this, we also ran exploratory analyses to assess these alternative pathways and gauge their comparative significance relative to varied LOC eating dimensions. Further, a prior analysis of the present sample examined whether the momentary negative affect‐binge eating association was mediated by negative urgency (i.e., a self‐regulatory process that overlaps with emotion dysregulation [*r* = 0.948 in the present study] and is defined as behaving impulsively while experiencing negative affect; Wonderlich et al. 2024). The present study extends this and other publications using this dataset (e.g., Peterson et al. 2020; Schaefer et al. 2020; Wonderlich et al. 2022, 2024) by assessing: (1) momentary mediational pathways that include positive affect as a predictor (vs. negative affect only); (2) different LOC eating outcomes (vs. binge eating only). The decision to examine the targeted momentary mediational associations in relation to different LOC eating dimensions, rather than a unidimensional LOC eating construct, is based on prevailing theoretical and conceptual knowledge, clinical observations, and existing between‐person (e.g., cross‐sectional) research (Latner et al. 2014; Stefano et al. 2016). These results of the present study can serve as an initial test of whether emotion dysregulation is a mechanism of action in theoretically supported associations between affect and LOC eating at the momentary level, and variations in these associations based on affect and LOC eating type.

## 2 Method

### 2.1 Participants and Procedures

Institutional review board approval was obtained from the participating institutions. All procedures were performed in accordance with the ethical standards in the 1964 Declaration of Helsinki and its amendments. Informed consent was obtained from all participants.

The present study used baseline EMA data from a multisite randomized controlled trial (NCT0204349) testing the efficacy of Integrative Cognitive‐Affective Therapy (ICAT‐BED) versus guided self‐help Cognitive Behavioral Therapy for Eating Disorders (CBTgsh). A detailed description of participant eligibility criteria and study procedures are reported in Peterson et al. (2020). Briefly, participants included 112 adults ages 18–65 with DSM‐5 BED. At baseline, participants completed five semi‐randomly prompted EMA surveys (wherein days were separated into equally spaced time windows and surveys were deployed at randomly prompted times within each window) plus one nightly survey on mobile devices daily for 7 days via the ReTAINE software. The EMA surveys were deployed between 8 a.m. and 10 p.m. each day. Five participants did not provide sufficient EMA data on measures of interest (i.e., no data on the lagged affect or emotion dysregulation variables in relation to LOC eating events), leaving an analytic sample of 107 participants. Participants without sufficient data did not differ from those in the analytic sample on baseline methodological (e.g., treatment group, treatment completion status), demographic (e.g., gender, BMI), or psychopathological factors (e.g., trait‐level ED pathology, trait‐level emotion dysregulation; see Data S1).

### 2.2 Measures

#### 2.2.1 Affect

Positive (determined, attentive, inspired, active) and negative (afraid, nervous, ashamed, sad, hostile) affect items from the Positive and Negative Affect Schedule (PANAS; Watson et al. 1988) and the Profile of Mood States (POMS; McNair et al. 1971) assessed participants' affect since their last EMA surveys (1 = *Not at all*, 5 = *Extremely*). Higher scores reflect greater positive and negative affect. The affect items were chosen based on pilot testing (Wonderlich et al. 2014) and prior research demonstrating that the assessed affect words exhibit robust associations with DEBs among eating disorder samples (Goldschmidt et al. 2012; Haedt‐Matt and Keel 2011; Wonderlich et al. 2022). The PANAS and POMS have exhibited good internal consistency, acceptable‐to‐good test–retest reliability, and good convergent validity with other affect measures among college, clinical, and community‐based samples (McNair et al. 1971; Watson et al. 1988). The factor structure of these constructs was confirmed in the present sample via multilevel confirmatory factor analyses (MCFAs; see Data S1).

#### 2.2.2 Emotion Dysregulation

The State‐Difficulties with Emotion Regulation Scale's (S‐DERS; Lavender et al. 2017) Modulate subscale assessed emotion dysregulation since participants' last EMA surveys (1 = *Not at all*, 5 = *Completely*). Higher scores reflect greater difficulties modulating momentary emotional and behavioral responses (example question: “Please indicate how much each statement applies to you right now…I am having difficulty controlling my behaviors”). The S‐DERS has exhibited good internal consistency and convergent validity with trait‐based emotion dysregulation measures among community‐based women (Lavender et al. 2017). The factor structure was confirmed via MCFA in the present sample following minor modifications (see Data S1).

#### 2.2.3 LOC Eating

To assess LOC eating dimensions, six items were adapted from established trait‐level eating disorder measures (Blomquist et al. 2014; Fairburn and Beglin 1994; Latner et al. 2014), the DSM‐5‐TR LOC eating operationalization (APA 2022), and prior ambulatory assessment research (e.g., Berg et al. 2017; Haedt‐Matt and Keel 2011). These dimensions include: (1) a general feeling of LOC while eating; (2) difficulties resisting eating; (3) difficulties stopping eating after starting; (4) feeling driven or compelled to eat; (5) not paying attention to one's eating behavior; (6) feeling disconnected while eating (e.g., numb, zoned out, on auto‐pilot). Participants endorsed the extent to which they engaged in each behavior since their last survey (1 = *Not at all*, 5 = *Very much*). Higher scores reflect greater LOC eating severity.

Participants were presented with the LOC eating items if they endorsed that they ate since their last survey. Sensitivity analyses showed that the overall pattern of results was consistent when data from assessments that followed eating episodes only were used, and those in which non‐eating and eating episodes were included (wherein non‐eating episodes were coded as 1, indicating that participants endorsed the extent that they engaged in each LOC eating type as “Not at all”). In the present study, LOC eating reports that followed eating episodes only were used to avoid introducing confounds.

### 2.3 Statistical Analyses

Statistical analyses were run using R 4.2.0. First, missing data analyses were run following recommended procedures and are shown in Data S1 (Enders 2022). Results indicated that the data were generally missing at random relative to the survey number of the day (1–6), but not other methodological (e.g., compliance, day in the study, treatment group), demographic (e.g., gender, BMI), or psychopathological factors (e.g., trait‐level ED pathology, trait‐level emotion dysregulation), nor were they suggested to be missing not at random. To adjust for this, the survey number of the day was controlled in all models (Enders 2022).

**FIGURE 1:**
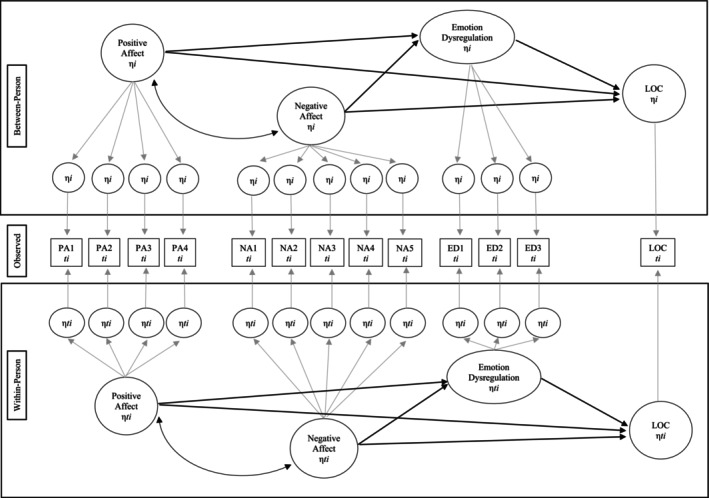
Conceptual model depicting the associations of interest that were examined in the assessed multilevel structural equation models. *i* = individual; ED = emotion dysregulation; LOC = loss of control eating; NA = negative affect; PA = positive affect; *t* = time; negative affect and positive affect were predictors, the emotion dysregulation factors were mediators, and (in separate models) each LOC eating behavior was the outcome; to capture the temporal sequencing of the targeted associations and establish temporal precedence that is helpful to examine when mediational associations are assessed, indicators for the latent factors were lagged twice for the predictors (affect) and once for the mediators (emotion dysregulation).

Second, factor structures were confirmed using MCFAs, as described above, and are reported in Data S1. Third, six two‐level (L) multilevel structural equation models (MSEMs) with a 1‐1‐1 structure were run using latent variable decomposition (i.e., a reflective modeling approach that is recommended for MSEMs, wherein the group [L2] effect is treated as an unobserved latent variable that is inferred from the observed [L1] data) to decrease bias in parameter estimates at L2 (Lüdtke et al. 2008). MSEMs were chosen, given the nature of the data (EMA, warranting multilevel analysis) and the present study's aim to examine momentary mediational associations (warranting a SEM‐based multilevel model that is designed to test mediational associations; Kline 2023). Momentary observations at L1 were nested within participants at L2. In the main analyses, negative affect and positive affect were modeled as predictors, emotion dysregulation as the mediator, and—in separate models—the LOC eating behaviors as outcomes. All models controlled for survey number of the day (given missing data analyses) and were estimated using maximum likelihood estimation (Enders 2022). See Figure 1 for a general model depiction.

Indicators for predictors' latent factors were lagged twice (time [*t*] − 2), indicators for mediators' latent factors were lagged once (*t* − 1), and outcomes were not lagged to appropriately assess mediational effects (Kline 2023). For time‐lagged variables, the last survey on the previous day was not lagged to the first survey on the next day, nor were nonconsecutive surveys (e.g., when surveys were missed). Direct effects from predictors to outcomes were included at both levels. Fixed slopes and random intercepts were modeled at both levels due to convergence difficulties when random slopes were added (because of the data structure's complexity).

While model building, evidence of poor fit was addressed by examining the sources of misfit (e.g., cross‐loadings, correlated residuals) and taking recommended actions (e.g., removing problematic items; Kline 2023). This occurred for the emotion dysregulation MCFA only (no MSEMs). One modification was made at a time to ensure that the impact of each could be gauged and was theoretically justified. Modifications were only made if the Akaike Information Criteria and sample‐size adjusted Bayesian Information Criteria decreased after each change (Kline 2023). Adequate‐to‐good fit was defined as Comparative Fit Index and Tucker Lewis Index > 0.90, Root Mean Square Error of Approximation < 0.08, and Standardized Root Mean Square Residuals < 0.08 (Kline 2023).

## 3 Results

Characteristics for the present sample (*N* = 107) are reported in Table 1. Participants responded to 3131 out of 4494 possible surveys, reflecting good compliance (70.00%). Most participants identified as women (*n* = 88, 82.24%) and White (*n* = 99, 92.52%). On average, participants were 39.87 (SD = 13.35) years old and had a BMI of 35.10 (SD = 8.67). Further, participants generally reported LOC eating at a moderate level of severity across the assessed dimensions, yet with high variability (all ranges = 1–5): (1) general feeling of losing control while eating (*M* = 2.26, SD = 1.32); (2) difficulties resisting eating (*M* = 2.79, SD = 1.42); (3) difficulties stopping eating after starting (*M* = 2.54, SD = 1.41); (4) feeling driven or compelled to eat (*M* = 2.88, SD = 1.38); (5) not paying attention to one's eating (*M* = 3.45, SD = 1.18); (6) feeling disconnected while eating (*M* = 2.19, SD = 1.26).

**TABLE 1:** Participant characteristics.

	*n* (%)	*M* (SD)
Baseline age		39.87 (13.35)
Baseline BMI		35.10 (8.67)
Baseline depressive symptoms (BDI total score)		15.00 (10.00)
Baseline eating disorder‐related clinical impairment (CIA total score)		23.61 (9.87)
Baseline emotion dysregulation (DERS total score)		89.26 (26.61)
Baseline eating disorder pathology (EDE global score)		2.69 (0.85)
Gender identity		
Men	18 (16.82%)	
Women	88 (82.24%)	
Transgender women	1 (0.93%)	
Transgender men	0	
Other gender identity	0	
Highest level of education		
8th grade education or less	0	
Some high school	1 (0.93%)	
Completed high school	6 (5.61%)	
Some college	21 (19.63%)	
Associate's degree	6 (5.61%)	
Bachelor's degree	29 (27.10%)	
Some graduate school	14 (13.08%)	
Graduate level degree	25 (23.36%)	
Not reported	5 (4.67%)	
Race/ethnicity		
White	99 (92.52%)	
Hispanic	1 (0.93%)	
Asian	1 (0.93%)	
Black	0	
American Indian or Alaska Native	0	
Not reported	6 (5.61%)	

*Note: N* = 107; additional participant characteristics are reported in Peterson et al. (2020).

Abbreviations: BDI = Beck's Depression Inventory; BMI = body mass index; CIA = Clinical Assessment of Impairment; DERS = Difficulties in Emotion Regulation Scale; EDE = Eating Disorder Examination.

### 3.1 Affect, Emotion Dysregulation, and LOC Eating

Results of the MSEMs that examined associations among negative and positive affect (predictors), emotion dysregulation (mediator), and different LOC eating dimensions (outcomes) are reported in Table 2. All MSEMs exhibited good fit (see Data S1) and did not require modifications.

**TABLE 2:** Multilevel structural equation models examining momentary associations among negative and positive affect, emotion dysregulation, and loss of control eating.

	Within‐person			Between‐person		
	*b* (SE)	*p*	*β*	*b* (SE)	*p*	*β*
Model 1: Negative and Positive Affect (X) ➔ Emotion Dysregulation (M) ➔ LOC: General Feeling of LOC While Eating (Y)						
Direct effects						
Negative affect ➔ Emotion dysregulation	**0.35 (0.06)**	**< 0.001**	**0.24**	**0.96 (0.10)**	**< 0.001**	**0.90**
Positive affect ➔ Emotion dysregulation	**−0.07 (0.03)**	**0.040**	**−0.08**	−0.02 (0.06)	0.725	−0.02
Positive affect ➔ Emotion dysregulation ➔ LOC eating	**0.28 (0.07)**	**< 0.001**	**0.13**	**0.82 (0.41)**	**0.048**	**0.68**
Negative affect ➔ LOC eating	−0.05 (0.12)	0.676	−0.02	−0.14 (0.44)	0.749	−0.11
Positive affect ➔ LOC eating	0.06 (0.07)	0.350	0.03	−0.02 (0.11)	0.871	−0.02
Indirect effects						
Negative affect ➔ Emotion dysregulation ➔ LOC eating	**0.10 (0.03)**	**0.001**	**0.03**	0.78 (0.40)	0.052	0.61
Positive affect ➔ Emotion dysregulation ➔ LOC eating	−0.02 (0.01)	0.070	−0.01	−0.02 (0.05)	0.728	−0.02
Model 2: Negative and Positive Affect (X) ➔ Emotion Dysregulation (M) ➔ LOC: Difficulties Resisting Eating (Y)						
Direct effects						
Negative affect ➔ Emotion dysregulation	**0.35 (0.06)**	**< 0.001**	**0.24**	**0.96 (0.10)**	**< 0.001**	**0.90**
Positive affect ➔ Emotion dysregulation	**−0.07 (0.03)**	**0.039**	**−0.08**	−0.02 (0.06)	0.731	−0.02
Positive affect ➔ Emotion dysregulation ➔ LOC eating	**0.16 (0.07)**	**0.017**	**0.08**	0.62 (0.49)	0.208	0.44
Negative affect ➔ LOC eating	0.05 (0.11)	0.629	0.02	−0.01 (0.53)	0.992	−0.004
Positive affect ➔ LOC eating	**0.14 (0.06)**	**0.022**	**0.08**	−0.10 (0.13)	0.445	−0.08
Indirect effects						
Negative affect ➔ Emotion dysregulation ➔ LOC eating	**0.06 (0.03)**	**0.026**	**0.02**	0.59 (0.47)	0.210	0.40
Positive affect ➔ Emotion dysregulation ➔ LOC eating	−0.01 (0.01)	0.122	−0.01	−0.01 (0.04)	0.740	−0.01
Model 3: Negative and Positive Affect (X) ➔ Emotion Dysregulation (M) ➔ LOC: Difficulties Stopping Eating After Starting (Y)						
Direct effects						
Negative affect ➔ Emotion dysregulation	**0.35 (0.06)**	**< 0.001**	**0.24**	**0.96 (0.10)**	**< 0.001**	**0.90**
Positive affect ➔ Emotion dysregulation	**−0.07 (0.03)**	**0.040**	**−0.08**	−0.02 (0.06)	0.730	−0.02
Positive affect ➔ Emotion dysregulation ➔ LOC eating	0.11 (0.07)	0.143	0.05	0.89 (0.46)	0.055	0.67
Negative affect ➔ LOC eating	0.05 (0.12)	0.671	0.02	−0.24 (0.49)	0.619	−0.17
Positive affect ➔ LOC eating	0.03 (0.07)	0.655	0.02	−0.06 (0.12)	0.631	−0.05
Indirect effects						
Negative affect ➔ Emotion dysregulation ➔ LOC eating	0.04 (0.03)	0.154	0.01	0.85 (0.45)	0.060	0.61
Positive affect ➔ Emotion dysregulation ➔ LOC eating	−0.01 (0.01)	0.233	−0.004	−0.02 (0.05)	0.733	−0.02
Model 4: Negative and Positive Affect (X) ➔ Emotion Dysregulation (M) ➔ LOC: Driven or Compelled to Eat (Y)						
Direct effects						
Negative affect ➔ Emotion dysregulation	**0.35 (0.06)**	**< 0.001**	**0.24**	**0.95 (0.10)**	**< 0.001**	**0.90**
Positive affect ➔ Emotion dysregulation	**−0.07 (0.03)**	**0.040**	**−0.08**	−0.02 (0.06)	0.719	−0.02
Positive affect ➔ Emotion dysregulation ➔ LOC eating	**0.27 (0.07)**	**< 0.001**	**0.14**	0.69 (0.49)	0.161	0.47
Negative affect ➔ LOC eating	−0.06 (0.11)	0.565	−0.02	−0.01 (0.52)	0.979	−0.01
Positive affect ➔ LOC eating	−0.01 (0.06)	0.918	−0.004	−0.08 (0.13)	0.558	−0.06
Indirect effects						
Negative affect ➔ Emotion dysregulation ➔ LOC eating	**0.09 (0.03)**	**0.001**	**0.03**	0.66 (0.47)	0.164	0.43
Positive affect ➔ Emotion dysregulation ➔ LOC eating	−0.02 (0.01)	0.067	−0.01	−0.01 (0.04)	0.727	−0.01
Model 5: Negative and Positive Affect (X) ➔ Emotion Dysregulation (M) ➔ LOC: Not Paying Attention to One's Eating (Y)						
Direct effects						
Negative affect ➔ Emotion dysregulation	**0.35 (0.06)**	**< 0.001**	**0.24**	**0.95 (0.10)**	**< 0.001**	**0.90**
Positive affect ➔ Emotion dysregulation	**−0.07 (0.03)**	**0.042**	**−0.08**	−0.03 (0.06)	0.666	−0.03
Positive affect ➔ Emotion dysregulation ➔ LOC eating	−0.01 (0.06)	0.858	−0.01	0.36 (0.40)	0.378	0.30
Negative affect ➔ LOC eating	0.06 (0.10)	0.569	0.02	−0.45 (0.43)	0.289	−0.36
Positive affect ➔ LOC eating	−0.07 (0.05)	0.196	−0.05	**−0.50 (0.12)**	**< 0.001**	**−0.48**
Indirect effects						
Negative affect ➔ Emotion dysregulation ➔ LOC eating	−0.004 (0.02)	0.858	−0.001	0.34 (0.38)	0.380	0.27
Positive affect ➔ Emotion dysregulation ➔ LOC eating	0.001 (0.004)	0.859	0.000	−0.01 (0.02)	0.689	−0.01
Model 6: Negative and Positive Affect (X) ➔ Emotion Dysregulation (M) ➔ LOC: Felt Disconnected While Eating (Y)						
Direct effects						
Negative affect ➔ Emotion dysregulation	**0.35 (0.06)**	**< 0.001**	**0.24**	**0.95 (0.10)**	**< 0.001**	**0.90**
Positive affect ➔ Emotion dysregulation	**−0.07 (0.03)**	**0.038**	**−0.08**	−0.02 (0.06)	0.723	−0.02
Positive affect ➔ Emotion dysregulation ➔ LOC eating	**0.25 (0.06)**	**< 0.001**	**0.14**	**1.00 (0.41)**	**0.014**	**0.77**
Negative affect ➔ LOC eating	0.03 (0.10)	0.786	0.01	−0.29 (0.43)	0.497	−0.21
Positive affect ➔ LOC eating	−0.03 (0.06)	0.614	−0.02	**−0.32 (0.11)**	**0.002**	**−0.29**
Indirect effects						
Negative affect ➔ Emotion dysregulation ➔ LOC eating	**0.09 (0.03)**	**0.001**	**0.03**	**0.95 (0.40)**	**0.017**	**0.69**
Positive affect ➔ Emotion dysregulation ➔ LOC eating	−0.02 (0.01)	0.065	−0.01	−0.02 (0.06)	0.725	−0.02

*Note*: Bold text is used to reflect significant effects, defined as *p* < 0.05; momentary observations at level 1 were nested within participants at level 2; indicators for the predictors' (negative affect, positive affect) latent factors were lagged twice, indicators for the mediators' (emotion dysregulation) latent factor were lagged once, and the dependent variable (LOC eating) was not lagged; latent variable decomposition was used to decrease bias in parameter estimates at level 2 (Lüdtke et al. 2008); all models were estimated using maximum likelihood estimation; all models controlled for survey number of the day (1–6) due to results of missing data analyses; model fit statistics were good and are reported in Data S1.

Abbreviation: LOC = Loss of control.

#### 3.1.1 Affect (X) ➔ Emotion Dysregulation (M)

Regarding the direct effects, across models, there were significant positive associations between the negative affect and emotion dysregulation latent factors at the within‐person (*β* = 0.24) and between‐person (*β* = 0.90) levels, and inverse associations between the positive affect and emotion dysregulation factors at the within‐person (*β* = −0.08)—but not between‐person—level. That is, at times when participants reported greater negative affect and less positive affect than they usually did, they subsequently reported greater emotion dysregulation at their next assessments (within‐person effects). Further, participants who generally reported greater negative affect than other participants also generally reported greater emotion dysregulation throughout the EMA period (between‐person effect).

#### 3.1.2 Emotion Dysregulation (M) ➔ LOC Eating (Y)

Direct effects between the emotion dysregulation and LOC eating factors varied based on the LOC eating dimension. Specifically, there were significant positive associations between the emotion dysregulation factors relative to the general sense of LOC while eating and feelings of disconnection (e.g., numb, zoned out, on auto‐pilot) while eating factors at both the within‐person (*β* = 0.13–0.14) and between‐person (*β* = 0.68–0.77) levels. The emotion dysregulation factor was also positively associated with the difficulties resisting eating and feeling driven or compelled to eat factors at the within‐person (*β* = 0.08–0.14)—but not between‐person—level, but not the difficulties stopping eating after starting or the not paying attention to one's eating factors at either level. That is, at times when participants reported greater emotion dysregulation than usual, they subsequently reported a greater general sense of LOC while eating, feeling more disconnected while eating, experiencing more difficulties resisting eating, and feeling more driven/compelled to eat at their next assessments. Further, participants who generally reported greater emotion dysregulation than other participants also generally reported a greater general sense of LOC while eating and feeling more disconnected while eating throughout the study.

#### 3.1.3 Indirect Effects

The emotion dysregulation factor mediated positive associations between the negative affect and feeling disconnected while eating factors at the within‐person (*β* = 0.03) and between‐person (*β* = 0.69) levels. There were also significant indirect effects at the within‐person—but not between‐person—level among the negative affect, emotion dysregulation, and (separately) general sense of LOC while eating, difficulties resisting eating, and feeling driven/compelled to eat factors (*β* = 0.02–0.03). Specifically, at times when participants reported greater negative affect than usual, they subsequently reported greater emotion dysregulation which, in turn, was associated with a greater feeling of disconnection while eating, a greater general sense of LOC while eating, more difficulties resisting eating, and feeling more driven/compelled to eat at their next assessments. Also, participants who generally reported greater negative affect than other participants generally reported greater emotion dysregulation and, in turn, feeling more disconnected while eating throughout the study. The emotion dysregulation factors did not mediate associations between the negative affect factors and either the difficulties stopping eating after starting or the not paying attention to one's eating factors, or the positive affect factors and any LOC eating factors, at the within‐person or between‐person levels.

### 3.2 Exploratory Analyses

Results of exploratory MSEMs that examined negative and positive affect as momentary mediators of momentary emotion dysregulation‐LOC eating dimension associations are reported in Data S1. Results generally aligned with the primary analyses in which momentary emotion dysregulation was modeled as a mediator of momentary associations between momentary affect‐LOC eating dimension associations. Specifically, in the exploratory analyses, indirect effects were significant for models involving momentary emotion dysregulation (predictor), negative affect (mediator), and four LOC eating dimensions (outcomes): general LOC eating, difficulties resisting eating, feeling driven/compelled to eat, and feeling disconnected while eating. In other words, at times when participants reported greater difficulties with momentary emotion dysregulation than usual, they subsequently reported greater negative affect which, in turn, was associated with a greater general sense of LOC while eating, more difficulties resisting eating, feeling more driven/compelled to eat, and a greater feeling of disconnection while eating at their next assessments. In contrast, the respective indirect effects were not significant in exploratory MSEMs when the difficulties stopping eating after starting and not paying attention to one's eating behaviors LOC eating dimensions were modeled as outcomes, nor were any pathways involving positive affect as a predictor or pathways at the between‐person level.

## 4 Discussion

Few studies have directly assessed mechanistic processes that are theorized to promote core disinhibited DEBs that characterize BED in the natural environment, such as emotion dysregulation. The present study used EMA to address this research gap by examining whether: (1) emotion dysregulation mediated associations between negative and positive affect and LOC eating at the within‐person (momentary) level; (2) these associations varied across distinct LOC eating dimensions. Results showed that greater momentary emotion dysregulation mediated momentary associations between greater negative affect and several LOC eating dimensions. However, notable variations in this pattern of results emerged based on the LOC eating dimension under consideration. Also, all momentary mediational pathways involving positive affect as a predictor were not significant. Collectively, these findings provide initial insight into theoretically supported mechanistic processes (Hawkins and Clement 1984; Heatherton and Baumeister 1991; Mason et al. 2021) that may be leveraged for testing as treatment targets in novel real‐time BED interventions and help improve BED research and theory refinement.

### 4.1 Variations Across LOC Eating Dimensions

The present results showed that when participants experienced a sequential worsening of negative affect and, in turn, emotion dysregulation over a day (modeled via time‐lagged MSEM indicators/variables), they subsequently reported more severe levels of certain LOC eating outcomes (general sense of LOC while eating, difficulties resisting eating, feeling driven or compelled to eat, feeling disconnected while eating), but not others (difficulties stopping eating after starting, not paying attention to one's eating). Exploratory analyses indicated that greater momentary negative affect similarly served as a momentary mediator of associations between greater momentary emotion dysregulation relative to the same four aforementioned LOC eating dimensions. This suggests that an adverse cycle was present in which bidirectional associations between greater negative affect and emotion dysregulation prompted greater reports of specific types of LOC eating. The present study was the first (to our knowledge) to use EMA to assess these momentary mediational associations, which limits comparisons with prior research. However, these results are consistent with and extend prior between‐person (cross‐sectional) research that has conceptualized LOC eating as a heterogeneous and dimensional construct with distinct behavioral and cognitive dimensions that have exhibited unique associations with eating disorder and general psychopathology at the between‐person (or trait) level (e.g., Latner et al. 2014; Stefano et al. 2016). These findings similarly align with and extend results from two initial studies that used ambulatory assessment methods to examine associations among affect, emotion dysregulation, and DEBs at the within‐person level of analysis among community‐based twin women (Mikhail et al. 2024) and young women with ED pathology (Romano et al. 2024), but not adults with BED. Specifically, these prior studies tested emotion dysregulation as a momentary mediator of associations between negative and positive affect relative to DEBs (Romano et al. 2024), and whether negative and positive affect served as daily or momentary mediators of daily or momentary associations between emotion dysregulation and DEBs (Mikhail et al. 2024; Romano et al. 2024). Notably, the present results serve as important extensions of these two prior studies, as neither prior study included adults with BED or examined how these within‐person mediational associations differentially mapped onto distinct LOC eating dimensions. Also, existing research that has used EMA to assess correlates of LOC eating in general—regardless of the targeted associations—has almost exclusively operationalized this construct as “a general sense of LOC while eating” using single survey items (e.g., Goldschmidt 2017; Haedt‐Matt and Keel 2011; Romano et al. 2024). The present results highlight the importance of moving beyond this general operationalization in future EMA research, and accounting for other clinically, diagnostically, and theoretically important facets of LOC eating that individuals can experience with varying degrees of severity on a moment‐to‐moment or day‐to‐day basis (APA 2022; Latner et al. 2014; Stefano et al. 2016; WHO 2019).

Although it is unclear why experiencing a sequential worsening of negative affect and, in turn, emotion dysregulation (or vice versa) over a day maps onto some LOC eating dimensions and not others, the present findings suggest that there may be certain stages of a LOC eating episode that may be precipitated by such a sequence. Specifically, these findings suggest that a progressive exacerbation of negative affect and emotion dysregulation over a day may more reliably predict whether people with BED will have difficulties ultimately resisting or otherwise thwarting the drive/compulsion to engage in LOC eating and will feel more dissociated during the episode (given the statistical significance of the momentary mediational associations targeting these LOC eating dimensions). Conversely, the pathway with negative affect and emotion dysregulation modeled as sequential momentary predictors of the difficulties stopping eating after starting dimension was not significant, indicating that there was greater variability in whether moment‐to‐moment changes in the two self‐regulatory factors promoted this LOC eating outcome. This finding suggests that there may be other intrapersonal, interpersonal, or context‐related factors that arise during a LOC eating episode that impact whether people with BED struggle to end the episode. For example, such variability may stem from others unexpectedly entering the room during a LOC eating episode that an individual wants to conceal (e.g., due to feelings of shame, guilt; APA 2022; O'Loghlen et al. 2022) or inconsistencies in whether individuals engage in adaptive or other maladaptive behaviors besides LOC eating as attempts to cope with heightened negative affect and/or emotion dysregulation (e.g., cognitive restructuring, substance misuse) on a given day. Future research is needed to corroborate these hypotheses and gauge their generalizability to other samples.

### 4.2 Positive Affect

It is noteworthy that all momentary pathways, including positive affect as a predictor of emotion dysregulation and, in turn, LOC eating dimensions, were not significant in the present study. To our knowledge, no prior research has used EMA to examine whether momentary emotion dysregulation mediates momentary associations between positive affect and LOC eating. However, the present results are consistent with proxy evidence from a daily diary study with community‐based twin women that showed that daily positive affect did not significantly mediate daily emotion (dys)regulation strategy use–binge eating associations (Mikhail et al. 2024). Although LOC eating was not assessed as an outcome or emotion dysregulation as a mediator in this prior study—which limits comparisons with the present findings—these data may collectively suggest that: (1) experiencing both low positive affect and elevated emotion dysregulation may not directly promote disinhibited DEBs in the natural environment; (2) additional factors may warrant consideration in these processes.

In particular, emerging research supporting positive affect dysregulation as a process that contributes to BED maintenance and disinhibited DEBs indicates that: (1) positive affect levels commonly decrease in the hours before and improve following individuals' engagement in disinhibited DEBs, suggesting that such dysregulation is a direct proximal contributor to these outcomes; and (2) positive affect may promote disinhibited DEBs in the natural environment via indirect pathways involving mechanisms that were not accounted for in the present study (Mason et al. 2021; Selby et al. 2019). For example, BED theory posits that the interaction between within‐ (e.g., momentary positive affect dysregulation) and between‐person (e.g., trait‐anhedonia) processes contribute to disinhibited DEBs maintenance among individuals with BED directly and indirectly through individuals' engagement in momentary maladaptive health behaviors (e.g., less physical activity, healthy eating, and social interactions) which, in turn, promote the maintenance of trait‐anhedonia (Mason et al. 2021). Alternatively, complementing Mason et al.'s (2021) theory, the mechanistic staging model of reward processing alterations that was designed for individuals with BED (Bodell and Racine 2023) posits that individuals with BED can exhibit changes in their desire to consume food and pleasure sensations that result from consuming food as their BED symptoms increase in severity. This process is believed to occur via changes in dysregulated reward‐related mechanisms, such as increases in food cravings and approach motivation, and decreases in consummatory pleasure (Bodell and Racine 2023). In future research, it may be helpful to examine whether accounting for these additional factors alters the present findings, and how momentary emotion dysregulation as a conceptually related construct may fit within these broader theoretical models.

### 4.3 Strengths, Limitations, and Future Directions

The present study has multiple strengths, including that it was the first to use EMA and advanced statistics to assess whether emotion dysregulation mediated associations between negative and positive affect and different LOC eating types at the momentary level among adults with BED. Findings from this study support how dynamic changes in transdiagnostic self‐regulatory factors are of central importance to understanding BED pathology and can differentially promote adults with BED's engagement in LOC eating in their natural environments based on the type of affect and LOC eating under consideration.

Despite these strengths, study limitations warrant attention. First, the present study included adults with BED who primarily identified as White women. It is unclear whether these findings generalize to youth, men, or nonclinical, other eating disorders, or marginalized populations. Future research would benefit from determining whether the results generalize to these other important groups. Second, some EMA items were adapted from trait‐level measures or selected from longer state‐level measures to compensate for the brevity of ambulatory assessment and limit participant burden, per recommendations (Shiffman et al. 2008). The latter resulted in select items being included for assessment (e.g., select affect, emotion dysregulation, and LOC eating items). Although validity‐related concerns stemming from measure adaptation are limited by the confirmation of measures' factor structures via MCFA, further research is warranted to establish additional psychometric properties and determine whether different factor structures may be applicable within other samples of participants (e.g., 3 vs. 4 LOC eating factors). Also, future research would benefit from assessing whether the present findings hold when other items are assessed (e.g., other affect terms, both positive and negative emotion dysregulation, other types of LOC eating). Third, the present findings strictly pertain to associations among affect, emotion dysregulation, and LOC eating based on the modulate emotion dysregulation dimension. As emotion dysregulation is a multidimensional construct (Gratz and Roemer 2004; Gross 1998a; Gross 1998b; Lavender et al. 2017), it will be helpful for future research to examine whether these results vary when other dimensions are considered.

The present findings have implications for measurement development and clinical practice. Regarding measure development, no prior research has developed and validated a LOC eating measure for administration via ambulatory assessment. The present findings suggest that LOC eating may warrant conceptualization as a multidimensional construct at the momentary level and, consequently, operationalization multidimensionally in a measure for EMA. Such measure development is important, given that LOC eating is a transdiagnostic DEB for individuals who not only experience BED but also other eating disorders and subclinical symptoms (APA 2022; Lavender et al. 2014; WHO 2019). Regarding practice, future research may leverage the present findings by developing just‐in‐time adaptive interventions (JITAIs) for adults with BED that target negative affect and emotion dysregulation. JITAIs deliver micro‐interventions to individuals via mobile devices at times when they are most vulnerable for engaging in harmful behaviors like LOC eating (Juarascio et al. 2018). The present findings suggest that it may be helpful for JITAIs to test whether prompting individuals to use adaptive emotion regulation strategies (e.g., cognitive restructuring, emotional acceptance) immediately after they report experiencing greater negative affect and/or difficulties with emotional and behavioral modulation than usual maps onto less LOC eating severity later that day and longer term.

Further, given the novelty of the assessed research questions, it is unclear whether the assessed sequencing of worse affective experiences, greater emotion dysregulation, and greater LOC eating over the course of a given day is more likely to manifest across a several‐hour period or a shorter timescale. Existing EMA data generally demonstrate that LOC eating and other types of disinhibited DEBs (e.g., binge eating) are generally reported by individuals later in the day, rather than in the morning or afternoon (Bottera and De Young 2024; Smyth et al. 2009). Such timing of these eating behaviors has been hypothesized to occur due to individuals experiencing a sequentially worsening mood and increasing difficulties with effectively regulating emotions and behaviors over the course of a day, among other factors (e.g., dietary restriction throughout the day; Bottera and De Young 2024; Smyth et al. 2009). This pattern suggests that the assessed mediational processes (involving affective experiences, emotion dysregulation, and LOC eating dimensions) are likely most pronounced when assessed over a several‐hour period. However, future research that directly assesses how these processes may manifest over varied timescales will serve as an important extension of the present study.

### 4.4 Conclusions

Results showed that greater emotion dysregulation mediated associations between greater negative affect and several LOC eating dimensions at the momentary level among adults with BED. However, notable variations in this pattern of results emerged based on the different LOC eating components. Also, all momentary mediational pathways involving positive affect as a predictor were not significant. Collectively, these findings provide initial support for theoretically supported mechanistic processes that may be leveraged for testing as treatment targets in novel real‐time interventions and can help enhance future BED research and theory refinement.

## Author Contributions

**Kelly A. Romano:** formal analysis, methodology, writing – original draft. **Carol B. Peterson:** conceptualization, investigation, writing – review and editing. **Glen Forester:** writing – review and editing. **Joseph A. Wonderlich:** writing – review and editing. **Stephen A. Wonderlich:** conceptualization, investigation, writing – review and editing. **Scott E. Engel:** conceptualization, investigation, writing – review and editing. **Ross D. Crosby:** conceptualization, investigation, writing – review and editing.

## Conflicts of Interest

The authors declare no conflicts of interest.

## Supporting information


**Data S1.** Supporting information.

## Data Availability

The data, material, and code that support the findings of this study are available from the corresponding author upon reasonable request.
