# Supplementary material for: Momentary Mediational Associations Among Affect, Emotion Dysregulation, and Different Types of Loss of Control Eating Among Adults With Binge Eating Disorder
Source: Int J Eat Disord. 2025 Mar 15;58(6):1072–84. doi: 10.1002/eat.24415 (PMC12138863; doi:10.1002/eat.24415)
Supplement: Supplementary file 1 — Data S1. Supporting information. [file EAT-58-1072-s001.docx]

Supplement

| Table S.1.  *Multi-Level Confirmatory Factor Analysis Model Fit Statistics* | | | | | | | | | |
| --- | --- | --- | --- | --- | --- | --- | --- | --- | --- |
|  |  |  |  |  |  | **RMSEA 90% Confidence Interval** | | **SRMR** | |
|  | ***χ*^2^ (df)** | ***p*** | **CFI** | **TLI** | **RMSEA** | **Lower Limit** | **Upper Limit** | **Within-Person Level** | **Between-Person Level** |
| Negative Affect | 69.690  (10) | <.001 | 0.960 | 0.919 | 0.064 | 0.050 | 0.079 | 0.037 | 0.028 |
| Positive Affect | 26.657  (4) | <.001 | 0.988 | 0.964 | 0.055 | 0.036 | 0.075 | 0.020 | 0.028 |
| Emotion Dysregulation | 0.034  (0) | 1.000 | 1.000 | 1.000 | 0.000 | 0.000 | 0.000 | 0.000 | 0.001 |
| *Note*. df = degrees to freedom; CFI = Comparative Fit Index; TLI = Tucker Lewis Index; RMSEA = Root Mean Square Error of Approximation; SRMR = Standardized Root Mean Square Residuals. | | | | | | | | | |

| Table S.2.  S*tandardized Covariances for the Loss of Control Eating Factors at the Momentary and Person Levels of Analysis* | | | | | | | |
| --- | --- | --- | --- | --- | --- | --- | --- |
|  | **Momentary-Level** | | | | | | |
| **Person-Level** |  | **General LOC Eating** | **Difficulties Resisting Eating** | **Difficulties Stopping Eating After Starting** | **Feeling Driven or Compelled to Eat** | **Not Paying Attention to One’s Eating Behavior** | **Feeling Disconnected While Eating (e.g., Numb, Zoned Out, On Auto-Pilot)** |
|  | **General LOC Eating** | - | 0.687 | 0.725 | 0.658 | 0.031 | 0.473 |
|  | **Difficulties Resisting Eating** | 0.853 | - | 0.729 | 0.722 | -0.023 | 0.382 |
|  | **Difficulties Stopping Eating After Starting** | 0.911 | 0.911 | - | 0.682 | 0.008 | 0.424 |
|  | **Feeling Driven or Compelled to Eat** | 0.839 | 0.934 | 0.892 | - | -0.022 | 0.416 |
|  | **Not Paying Attention to One’s Eating Behavior** | -0.023 | -0.189 | -0.058 | -0.095 | - | 0.161 |
|  | **Feeling Disconnected While Eating (e.g., Numb, Zoned Out, On Auto-Pilot)** | 0.658 | 0.539 | 0.595 | 0.619 | 0.413 | - |
| *Note.* LOC = loss of control; standardized covariances at the momentary-level (Level 1) are reported above the horizontal line and those at the person-level (Level 2) are reported below the horizontal line. | | | | | | | |

| Table S.3  *Model Fit Statistics for the Multilevel Structural Equation Models Examining Momentary Associations among Negative and Positive Affect (Predictors), Emotion Dysregulation (Mediator), and Loss of Control Eating Dimensions (Outcomes)* | | | | | | | | |
| --- | --- | --- | --- | --- | --- | --- | --- | --- |
| **Outcome** | **χ^2^ (df)** | ***p*** | **CFI** | **TLI** | **RMSEA** | **RMSEA 90% Confidence Interval** | **SRMR Within-Person Level** | **SRMR Between-Person Level** |
| General Feeling of LOC While Eating | 363.627 (132) | <.001 | 0.947 | 0.932 | 0.039 | 0.034, 0.044 | 0.045 | 0.053 |
| Difficulties Resisting Eating | 352.795 (132) | <.001 | 0.949 | 0.935 | 0.038 | 0.033, 0.043 | 0.045 | 0.052 |
| Difficulties Stopping Eating After Starting | 355.153 (132) | <.001 | 0.948 | 0.934 | 0.038 | 0.033, 0.043 | 0.045 | 0.052 |
| Driven or Compelled to Eat | 347.037 (132) | <.001 | 0.950 | 0.936 | 0.037 | 0.033, 0.042 | 0.045 | 0.051 |
| Not Paying Attention to One’s Eating | 347.720 (132) | <.001 | 0.950 | 0.936 | 0.038 | 0.033, 0.042 | 0.045 | 0.051 |
| Felt Disconnected While Eating | 353.502 (132) | <.001 | 0.949 | 0.935 | 0.038 | 0.033, 0.043 | 0.045 | 0.052 |
| *Note.* LOC = loss of control; df = degrees of freedom; CFI = Comparative Fit Index; TLI = Tucker Lewis Index; RMSEA = Root Mean Square Error of Approximation; SRMR = Standardized Root Mean Square Residuals; all models included negative affect and positive affect factors as predictors, the emotion dysregulation factor as a mediator, and the loss of control eating items shown in column 1 as outcomes. | | | | | | | | |

| Table S.4.  *Exploratory Multilevel Structural Equation Models Examining Momentary Associations among Emotion Dysregulation (Predictor), Negative and Positive Affect (Mediators), and Loss of Control Eating Dimensions (Outcomes)* | | | | | | |
| --- | --- | --- | --- | --- | --- | --- |
|  | **Within-Person** | | | **Between-Person** | | |
|  | ***b (SE)*** | ***p*** | **β** | ***b (SE)*** | ***p*** | **β** |
| **Model 1: Emotion Dysregulation (X) 🡪 Negative and Positive Affect (Ms) 🡪 LOC:** **General Feeling of LOC While Eating (Y)** | | | | | | |
| **Direct Effects** |  |  |  |  |  |  |
| Emotion Dysregulation 🡪 Negative Affect | **0.352 (0.033)** | **<.001** | **0.456** | **0.735 (0.079)** | **<.001** | **0.836** |
| Emotion Dysregulation 🡪 Positive Affect | -0.036 (0.045) | .419 | -0.031 | -0.224 (0.129) | .081 | -0.193 |
| Negative Affect 🡪 LOC Eating | **0.346 (0.129)** | **.007** | **0.117** | 0.259 (0.320) | .419 | 0.197 |
| Positive Affect 🡪 LOC Eating | -0.066 (0.073) | .370 | -0.033 | -0.002 (0.112) | .987 | -0.002 |
| Emotion Dysregulation 🡪 LOC Eating | -0.047 (0.090) | .599 | -0.021 | 0.429 (0.286) | .134 | 0.371 |
| **Indirect Effects** |  |  |  |  |  |  |
| Emotion Dysregulation 🡪 Negative Affect 🡪 LOC Eating | **0.122 (0.046)** | **.008** | **0.053** | 0.190 (0.234) | .417 | 0.165 |
| Emotion Dysregulation 🡪 Positive Affect 🡪 LOC Eating | 0.002 (0.004) | .548 | 0.001 | 0.000 (0.025) | .987 | 0.000 |
| **Model 2: Emotion Dysregulation (X) 🡪 Negative and Positive Affect (Ms) 🡪 LOC:** **Difficulties Resisting Eating (Y)** | | | | | | |
| **Direct Effects** |  |  |  |  |  |  |
| Emotion Dysregulation 🡪 Negative Affect | **0.352 (0.033)** | **<.001** | **0.456** | **0.734 (0.079)** | **<.001** | **0.835** |
| Emotion Dysregulation 🡪 Positive Affect | -0.036 (0.045) | .415 | -0.032 | -0.224 (0.129) | .082 | -0.193 |
| Negative Affect 🡪 LOC Eating | **0.249 (0.122)** | **.041** | **0.088** | 0.346 (0.374) | .354 | 0.225 |
| Positive Affect 🡪 LOC Eating | -0.003 (0.070) | .961 | -0.002 | -0.072 (0.133) | .588 | -0.062 |
| Emotion Dysregulation 🡪 LOC Eating | -0.086 (0.086) | .314 | -0.040 | 0.278 (0.333) | .405 | 0.205 |
| **Indirect Effects** |  |  |  |  |  |  |
| Emotion Dysregulation 🡪 Negative Affect 🡪 LOC Eating | **0.088 (0.043)** | **.043** | **0.040** | 0.254 (0.274) | .354 | 0.188 |
| Emotion Dysregulation 🡪 Positive Affect 🡪 LOC Eating | 0.000 (0.003) | .961 | 0.000 | 0.016 (0.031) | .601 | 0.012 |
| **Model 3: Emotion Dysregulation (X) 🡪 Negative and Positive Affect (Ms) 🡪 LOC:** **Difficulties Stopping Eating After Starting (Y)** | | | | | | |
| **Direct Effects** |  |  |  |  |  |  |
| Emotion Dysregulation 🡪 Negative Affect | **0.353 (0.033)** | **<.001** | **0.456** | **0.734 (0.079)** | **<.001** | **0.835** |
| Emotion Dysregulation 🡪 Positive Affect | -0.036 (0.045) | .414 | -0.032 | -0.224 (0.129) | .082 | -0.192 |
| Negative Affect 🡪 LOC Eating | 0.064 (0.127) | .612 | 0.022 | 0.250 (0.350) | .475 | 0.173 |
| Positive Affect 🡪 LOC Eating | -0.028 (0.073) | .702 | -0.014 | -0.061 (0.124) | .624 | -0.056 |
| Emotion Dysregulation 🡪 LOC Eating | 0.020 (0.090) | .828 | 0.009 | 0.391 (0.312) | .210 | 0.308 |
| **Indirect Effects** |  |  |  |  |  |  |
| Emotion Dysregulation 🡪 Negative Affect 🡪 LOC Eating | 0.023 (0.045) | .611 | 0.010 | 0.183 (0.256) | .474 | 0.145 |
| Emotion Dysregulation 🡪 Positive Affect 🡪 LOC Eating | 0.001 (0.003) | .728 | 0.000 | 0.014 (0.029) | .633 | 0.011 |
| **Model 4: Emotion Dysregulation (X) 🡪 Negative and Positive Affect (Ms) 🡪 LOC:** **Driven or Compelled to Eat (Y)** | | | | | | |
| **Direct Effects** |  |  |  |  |  |  |
| Emotion Dysregulation 🡪 Negative Affect | **0.352 (0.033)** | **<.001** | **0.456** | **0.735 (0.079)** | **<.001** | **0.835** |
| Emotion Dysregulation 🡪 Positive Affect | -0.036 (0.044) | .417 | -0.032 | -0.225 (0.129) | .080 | -0.194 |
| Negative Affect 🡪 LOC Eating | **0.299 (0.119)** | **.012** | **0.109** | 0.433 (0.369) | .240 | 0.273 |
| Positive Affect 🡪 LOC Eating | -0.108 (0.068) | .114 | -0.058 | -0.062 (0.133) | .641 | -0.052 |
| Emotion Dysregulation 🡪 LOC Eating | -0.035 (0.083) | .673 | -0.017 | 0.280 (0.330) | .396 | 0.200 |
| **Indirect Effects** |  |  |  |  |  |  |
| Emotion Dysregulation 🡪 Negative Affect 🡪 LOC Eating | **0.105 (0.043)** | **.013** | **0.050** | 0.318 (0.271) | .240 | 0.228 |
| Emotion Dysregulation 🡪 Positive Affect 🡪 LOC Eating | 0.004 (0.005) | .470 | 0.002 | 0.014 (0.031) | .649 | 0.010 |
| **Model 5: Emotion Dysregulation (X) 🡪 Negative and Positive Affect (Ms) 🡪 LOC:** **Not Paying Attention to One’s Eating (Y)** | | | | | | |
| **Direct Effects** |  |  |  |  |  |  |
| Emotion Dysregulation 🡪 Negative Affect | **0.353 (0.033)** | **<.001** | **0.456** | **0.735 (0.079)** | **<.001** | **0.833** |
| Emotion Dysregulation 🡪 Positive Affect | -0.036 (0.045) | .414 | -0.032 | -0.222 (0.127) | .081 | -0.193 |
| Negative Affect 🡪 LOC Eating | -0.203 (0.104) | .051 | -0.084 | -0.231 (0.306) | .451 | -0.180 |
| Positive Affect 🡪 LOC Eating | -0.081 (0.060) | .178 | -0.050 | **-0.449 (0.115)** | **<.001** | **-0.455** |
| Emotion Dysregulation 🡪 LOC Eating | 0.095 (0.074) | .200 | 0.051 | 0.159 (0.275) | .563 | 0.140 |
| **Indirect Effects** |  |  |  |  |  |  |
| Emotion Dysregulation 🡪 Negative Affect 🡪 LOC Eating | -0.072 (0.037) | .054 | -0.038 | -0.170 (0.226) | .453 | -0.150 |
| Emotion Dysregulation 🡪 Positive Affect 🡪 LOC Eating | 0.003 (0.004) | .483 | 0.002 | 0.100 (0.062) | .110 | 0.088 |
| **Model 6: Emotion Dysregulation (X) 🡪 Negative and Positive Affect (Ms) 🡪 LOC:** **Felt Disconnected While Eating (Y)** | | | | | | |
| **Direct Effects** |  |  |  |  |  |  |
| Emotion Dysregulation 🡪 Negative Affect | **0.353 (0.033)** | **<.001** | **0.457** | **0.732 (0.080)** | **<.001** | **0.834** |
| Emotion Dysregulation 🡪 Positive Affect | -0.037 (0.045) | .407 | -0.032 | -0.221 (0.129) | .087 | -0.190 |
| Negative Affect 🡪 LOC Eating | **0.215 (0.109)** | **.048** | **0.086** | 0.468 (0.309) | .130 | 0.330 |
| Positive Affect 🡪 LOC Eating | -0.116 (0.062) | .062 | -0.069 | **-0.304 (0.109)** | **.005** | **-0.284** |
| Emotion Dysregulation 🡪 LOC Eating | 0.131 (0.077) | .088 | 0.067 | 0.289 (0.274) | .291 | 0.232 |
| **Indirect Effects** |  |  |  |  |  |  |
| Emotion Dysregulation 🡪 Negative Affect 🡪 LOC Eating | **0.076 (0.039)** | **.049** | **0.039** | 0.343 (0.226) | .129 | 0.275 |
| Emotion Dysregulation 🡪 Positive Affect 🡪 LOC Eating | 0.004 (0.006) | .445 | 0.002 | 0.067 (0.045) | .138 | 0.054 |
| *Note.* LOC = Loss of control; bold text is used to reflect significant effects, defined as *p* < .05; momentary observations at level 1 were nested within participants at level 2; indictors for the predictors’ (negative affect, positive affect) latent factors were lagged twice, indicators for the mediators’ (emotion dysregulation) latent factor were lagged once, and the dependent variable (LOC eating) was not lagged; latent variable decomposition was used to decrease bias in parameter estimates at level 2 (Lüdtke et al., 2008); all models were estimated using maximum likelihood estimation; all models controlled for survey number of the day (1-6) due to results of missing data analyses. | | | | | | |

| Table S.5.  *Model Fit Statistics for the Exploratory Multilevel Structural Equation Models Examining Momentary Associations among Emotion Dysregulation (Predictor), Negative and Positive Affect (Mediators), and Loss of Control Eating Dimensions (Outcomes)* | | | | | | | | |
| --- | --- | --- | --- | --- | --- | --- | --- | --- |
| **Outcome** | **χ^2^ (df)** | ***p*** | **CFI** | **TLI** | **RMSEA** | **RMSEA 90% Confidence Interval** | **SRMR Within-Person Level** | **SRMR Between-Person Level** |
| General Feeling of LOC While Eating | 350.081  (132) | <.001 | 0.949 | 0.935 | 0.038 | 0.033, 0.043 | 0.048 | 0.059 |
| Difficulties Resisting Eating | 350.933  (132) | <.001 | 0.949 | 0.934 | 0.038 | 0.033, 0.043 | 0.049 | 0.058 |
| Difficulties Stopping Eating After Starting | 353.435  (132) | <.001 | 0.948 | 0.934 | 0.038 | 0.033, 0.043 | 0.049 | 0.059 |
| Driven or Compelled to Eat | 346.492  (132) | <.001 | 0.950 | 0.936 | 0.037 | 0.033, 0.042 | 0.049 | 0.054 |
| Not Paying Attention to One’s Eating | 339.801  (132) | <.001 | 0.951 | 0.937 | 0.037 | 0.032, 0.042 | 0.048 | 0.055 |
| Felt Disconnected While Eating | 367.887  (132) | <.001 | 0.945 | 0.930 | 0.039 | 0.035, 0.044 | 0.049 | 0.057 |
| *Note.* LOC = loss of control; df = degrees of freedom; CFI = Comparative Fit Index; TLI = Tucker-Lewis Index; RMSEA = Root Mean Square Error of Approximation; SRMR = Standardized Root Mean Square Residual; all models included negative affect and positive affect factors as predictors, the emotion dysregulation factor as a mediator, and the loss of control eating items shown in column 1 as outcomes. | | | | | | | | |

| Table S.6  *Results of Chi Square Tests and T-Tests that Examined Differences between Values on Constructs that were Measured at Baseline for Participants with Sufficient Data to be Included in the Analytic Sample (n=107) and Participants without Relevant Data (n=5)* | | | | |
| --- | --- | --- | --- | --- |
| **Construct Assessed at Baseline** | ***χ^2^* or *t*** | ***p*** | ***Cramer’s V* or *Cohen’s d*** | ***p* or 95% Confidence Interval** |
| Treatment Group (ICAT-BED versus CBTgsh) | 0.306 | .580 | 0.053 | .580 |
| Treatment Completion Status | 5.280 | .071 | 0.219 | .071 |
| Gender | 0.575 | .750 | 0.072 | .750 |
| Age | -1.009 | .315 | -0.591 | -1.740, 0.561 |
| Highest Level of Education | -0.276 | .783 | -0.162 | -1.309, 0.986 |
| Baseline Body Mass Index | -1.106 | .271 | -0.563 | -1.562, 0.439 |
| Baseline Beck Depression Inventory Total Score | 1.257 | .212 | 0.736 | -0.418, 1.886 |
| Baseline Clinical Impairment Assessment for Eating Disorders Total Score | -0.041 | .967 | -0.024 | -1.171, 1.123 |
| Baseline Difficulties in Emotion Regulation Scale Total Score | 0.775 | .440 | 0.453 | -0.696, 1.601 |
| Baseline Eating Disorder Examination Global Score | -0.468 | .641 | -0.238 | -1.236, 0.761 |
| *Note.* ICAT-BED = integrated cognitive affective therapy for binge eating disorder; CBTgsh = cognitive behavioral therapy guided self-help; *χ^2^* difference test and Cramer’s V values were computed for baseline constructs that were categorical variables, and independent samples t-tests and Cohen’s *d* values (with 95% confidence intervals that depicted the significance [or lack thereof] of Cohen’s *d,* with significant values defined as those in which the 95% confidence interval did not contain 0) were computed for baseline constructs that were continuous variables. | | | | |

**Missing Data Analyses**

Missing data analyses were run following recommended procedures (Enders, 2022). In line with these recommended procedures, whether the data were missing at random (i.e., which is evident when missingness is dependent on observed values in a dataset) was assessed by creating missingness indicators that reflected whether (coded as 1) or not (coded as 0) data for the EMA-assessed constructs of interest in the present study were missing. These missingness indicators were then used as outcome variables in multilevel models (to account for the nested nature of the data structure) that examined whether missing EMA responses were meaningfully and influentially linked to various methodological factors, as well as demographic and psychological factors that were assessed at baseline. Meaningful/influential covariates were determined using Cohen’s (1988) recommendations for small (*R*^2^ = 0.02), medium (*R*^2^ = 0.13), and large (*R*^2^ = 0.26) effects. Effects that were small to medium sized or larger (i.e., *R*^2^ ≥ 0.03), were considered influential. Effect sizes were used to identify influential correlates of missingness rather than p-values, as p-values are sample size dependent. Covariates that were identified as influential correlates of missingness (i.e., suggesting that the data were missing at random) were then included directly within the assessed statistical models.

As shown in full in Table S.7. below, effect sizes ranged from trivial to small in magnitude across the vast majority of models for all missingness tests. However, the data were generally shown to be missing at random relative to the survey number of the day (1-6; *R*^2^s = 0.029), but not other methodological (e.g., compliance, day in the study, treatment group), baseline demographic (e.g., gender, BMI), or baseline psychopathological factors (e.g., trait-level ED pathology, trait-level emotion dysregulation). To adjust for this, survey number of the day was controlled in all models (Enders, 2022).

Data were not suggested to be missing not at random (i.e., when missingness is dependent on psychological factors that are linked to the targeted population, which in this case would be suggested by eating disorder-related psychopathology). This was determined via the trivial associations that were identified for associations between baseline Eating Disorder Examination Global Scores (*R*^2^s = 0) and Clinical Impairment Assessment for Eating Disorders Total Scores (*R*^2^s = 0). If such analyses provided initial evidence that the data were missing not at random, additional analyses (e.g., sensitivity analyses, pattern mixture models) would have been warranted.

| Table S.7  *Results of Missing Data Analyses that Examined Whether Missing Data (Coded as 1), Versus Observed Data (Coded as 0), Were Associated with Methodological, Demographic, and Psychological Factors* | | | | | | |
| --- | --- | --- | --- | --- | --- | --- |
|  | **Missingness Indicator for General Feeling of LOC While Eating** | **Missingness Indicator for Difficulties Resisting Eating** | **Missingness Indicator for Difficulties Stopping Eating After Starting** | **Missingness Indicator for Driven or Compelled to Eat** | **Missingness Indicator for Not Paying Attention to One’s Eating** | **Missingness Indicator for Felt Disconnected While Eating** |
|  | ***R*^2^** | ***R*^2^** | ***R*^2^** | ***R*^2^** | ***R*^2^** | ***R*^2^** |
| **Methodological Covariates** | | | | | | |
| Day Number in the Study | 0.002 | 0.002 | 0.002 | 0.002 | 0.002 | 0.002 |
| Treatment Group (ICAT-BED vs. CBTgsh) | 0.000 | 0.000 | 0.000 | 0.000 | 0.000 | 0.000 |
| EMA Survey Number of the Day (1-6) | 0.029 | 0.029 | 0.029 | 0.029 | 0.029 | 0.029 |
| Compliance | 0.000 | 0.000 | 0.000 | 0.000 | 0.000 | 0.000 |
| Study Wave Number (Baseline = 0, Post-Treatment = 1, Follow-Up= 2) | 0.002 | 0.002 | 0.002 | 0.002 | 0.002 | 0.002 |
| **Baseline Demographic Covariates** | | | | | | |
| Age | 0.002 | 0.002 | 0.002 | 0.002 | 0.002 | 0.002 |
| Gender (0 = Male, 1 = Female) | 0.002 | 0.002 | 0.002 | 0.002 | 0.002 | 0.002 |
| Income Level | 0.000 | 0.000 | 0.000 | 0.000 | 0.000 | 0.000 |
| Body Mass Index | 0.002 | 0.002 | 0.002 | 0.002 | 0.002 | 0.002 |
| Race/Ethnicity (Non-White = 0, White = 1) | 0.000 | 0.000 | 0.000 | 0.000 | 0.000 | 0.000 |
| **Psychological Constructs** | | | | | | |
| Baseline Difficulties in Emotion Dysregulation Total Score | 0.000 | 0.000 | 0.000 | 0.000 | 0.000 | 0.000 |
| Baseline Beck Depression Inventory Total Score | 0.000 | 0.000 | 0.000 | 0.000 | 0.000 | 0.000 |
| Baseline Clinical Impairment Assessment for Eating Disorders Total Score | 0.000 | 0.000 | 0.000 | 0.000 | 0.000 | 0.000 |
| Baseline Eating Disorder Examination Global Score | 0.000 | 0.000 | 0.000 | 0.000 | 0.000 | 0.000 |
| *Note.* LOC = loss of control; ICAT-BED = integrated cognitive affective therapy for binge eating disorder; CBTgsh = cognitive behavioral therapy guided self-help; EMA = ecological momentary assessment; missingness indicators reflected whether data for the constructs of interest in the present study had missing data (coded as 1) or not (coded as 0) are shown in row 2; covariates that were assessed as correlates of missing data for the assessed constructs of interest are shown in column 1; Meaningful/influential covariates were determined using Cohen’s (1988) recommendations for small (*R*^2^ = 0.02), medium (*R*^2^ = 0.13), and large (*R*^2^ = 0.26) effects. Effects that were small to medium sized or larger (i.e., *R*^2^ ≥ 0.03), were considered influential. | | | | | | |
